# Short-term Safety and Quality of Life Outcomes Following Radioembolization in Primary and Secondary Liver Tumours: a Multi-centre Analysis of 200 Patients in France

**DOI:** 10.1007/s00270-020-02643-x

**Published:** 2020-09-25

**Authors:** Romaric Loffroy, Maxime Ronot, Michel Greget, Antoine Bouvier, Charles Mastier, Christian Sengel, Lambros Tselikas, Dirk Arnold, Geert Maleux, Jean-Pierre Pelage, Olivier Pellerin, Bora Peynircioglu, Bruno Sangro, Niklaus Schaefer, María Urdániz, Nathalie Kaufmann, José Ignacio Bilbao, Thomas Helmberger, Valérie Vilgrain, On behalf of the CIRT-FR Principal Investigators, Gilles Piana, Julien Frandon, Jean-Pierre Tasu, Hicham Kobeiter

**Affiliations:** 1 Department of Vascular and Interventional Radiology, Image-Guided Therapy Center, CHU Dijon Bourgogne, François-Mitterrand University Hospital, 14 Rue Gaffarel, 21000 Dijon, France; 2 Department of Radiology, Université de Paris, Hôpital Beaujon APHP, and CRI, INSERM 1149, Paris, France; 3 Service d’Imagerie interventionnelle, Hôpitaux Universitaires de Strasbourg, 1 Avenue Moliere, 67000 Strasbourg, France; 4 Department of Radiology, Angers University Hospital, 4 Rue Larrey, 49933 Angers, France; 5 Interventional Radiology, Centre Léon Bérard, 28 Prom. Léa Et Napoléon Bullukian, 69008 Lyon, France; 6 Interventional Radiology, Centre Hospitalier Universitaire de Grenoble, Boulevard de la Chantourne, 38100 Grenoble, France; 7 Interventional Radiology, Département d’anesthésie Chirurgie et Interventionel (DACI), Gustave Roussy, Université Paris-Saclay, Villejuif, France; 8 Oncology and Hematology, Asklepios Tumorzentrum Hamburg, AK Altona, Paul-Ehrlich-Str. 1, 22763 Hamburg, Germany; 9 Radiology, Universitair Ziekenhuis Leuven, Herestraat 49, 3000 Leuven, Belgium; 10 Department of Diagnostic Imaging and Interventional Radiology, Caen University and Medical Center, avenue de la Cote de Nacre, 14033 Caen, France; 11 Interventional Radiology Department, Hôpital Européen Georges Pompidou, 20 rue Leblanc, 75015 Paris, France; 12 Department of Radiology, School of Medicine, Hacettepe University, Sihhiye Campus, 06100 Ankara, Turkey; 13 Clínica Universidad de Navarra, IDISNA and CIBEREHD, Liver Unit, Avda. Pio XII 36, 31008 Pamplona, Spain; 14 Service de médecine nucléaire Et Imagerie moléculaire, Centre Hospitalier Universitaire de Lausanne, Rue du Bugnon 46, CH-1011 Lausanne, Switzerland; 15 Clinical Research Department, Cardiovascular and Interventional Radiological Society of Europe, Neutorgasse 9, 1010 Vienna, Austria; 16 Interventional Radiology, Clínica Universidad de Navarra, Avenida Pio XII, no 36, 31008 Pamplona, Spain; 17 Department of Radiology, Neuroradiology and Minimal-Invasive Therapy, Klinikum Bogenhausen, Englschalkinger Str. 77, 81925 Munich, Germany

**Keywords:** Radioembolization, Transarterial radioembolization, Yttrium-90, SIR-spheres, Interim analysis, SIRT

## Abstract

**Purpose:**

Radioembolization has emerged as a treatment modality for patients with primary and secondary liver tumours. This observational study CIRT-FR (CIRSE Registry for SIR-Spheres Therapy in France) aims to evaluate real-life clinical practice on all patients treated with transarterial radioembolization (TARE) using SIR-Spheres yttrium-90 resin microspheres in France. In this interim analysis, safety and quality of life data are presented. Final results of the study, including secondary effectiveness outcomes, will be published later. Overall, CIRT-FR is aiming to support French authorities in the decision making on reimbursement considerations for this treatment.

**Methods:**

Data on patients enrolled in CIRT-FR from August 2017 to October 2019 were analysed. The interim analysis describes clinical practice, baseline characteristics, safety (adverse events according to CTCTAE 4.03) and quality of life (according to EORTC QLQ C30 and HCC module) aspects after TARE.

**Results:**

This cohort included 200 patients with hepatocellular carcinoma (114), metastatic colorectal cancer (mCRC; 38) and intrahepatic cholangiocarcinoma (33) amongst others (15). TARE was predominantly assigned as a palliative treatment (79%). 12% of patients experienced at least one adverse event in the 30 days following treatment; 30-day mortality was 1%. Overall, global health score remained stable between baseline (66.7%), treatment (62.5%) and the first follow-up (66.7%).

**Conclusion:**

This interim analysis demonstrates that data regarding safety and quality of life generated by randomised-controlled trials is reflected when assessing the real-world application of TARE.

**Trial Registration:**

Clinical Trials.gov NCT03256994.

**Electronic supplementary material:**

The online version of this article (10.1007/s00270-020-02643-x) contains supplementary material, which is available to authorized users.

## Introduction

Transarterial radioembolization (TARE), also known as selective internal radiation therapy (SIRT), with SIR-Spheres Y-90 resin microspheres, is an endovascular procedure included within the interventional oncologic armamentarium to treat primary and secondary liver tumours [1–7].

CIRT-FR (CIRSE Registry for SIR-Spheres Therapy in France; NCT03256994) was developed as a France-only adaptation of the pan-European, prospective observational study CIRT (NCT02305459) [8]. While CIRT [9] had only collected data from one French hospital, CIRT-FR was open to all French centres and designed to exhaustively include as many patients in France as possible. Under the condition that the present study was conducted in order to collect data on the real-life clinical application of TARE with SIR-Spheres® in France, reimbursement for liver-only metastatic colorectal cancer (mCRC) was granted for 5 years by the French national health authorities (Haute Autorité de Santé, HAS) in March 2017.^1^ In March 2019, based on the results of the phase III randomized controlled trials SARAH [10] and SIRveNIB [11], HAS extended reimbursement for patients with intermediate or advanced hepatocellular carcinoma (HCC^2^). Final data from this observational study will support French authorities in the decision making on reimbursement considerations for TARE beyond 2022.

This publication is the 200-patient interim analysis of the prospective, post-market, observational study CIRT-FR, with the objective to report on patient characteristics, treatment details as well as safety and health-related quality of life.

The primary objective was to observe the real-life clinical application of TARE with SIR-Spheres Y-90 resin microspheres in the context of the patients’ continuum of care (please refer to Table 1). Secondary objectives were to assess safety, toxicity, quality of life, technical considerations and diagnosis and treatment-related considerations, such as intention of treatment, prior and post-TARE interventional procedures and/or systemic therapy.

**Table 1:** Treatment intent (Primary outcome measurement)

Primary endpoint	HCC *n* = 114 (%)	ICC *n* = 33 (%)	mCRC *n* = 38 (%)
(A) First-line TARE treatment with or without concomitant systemic therapy	43 (37.7)	18 (54.5)	–
(B) Second or subsequent line TARE treatment with or without concomitant systemic therapy after previous first-line systemic therapy, including salvage therapy when no other systemic therapies used alone are likely to be efficacious	7 (6.1)	8 (24.2)	20 (52.6)
(C) TARE treatment with or without concomitant systemic therapy after previous interventional liver-directed procedures or liver surgery	50 (43.8)	1 (3.0)	2 (5.2)
(D) Addition of TARE to systemic therapy (any line) or to any other treatment (e.g. ablation) intended as part of a multimodal curative therapy with any of the following objectives: resectability and/or ablative therapy and/or transplantation	6 (5.3)	3 (9.1)	2 (5.2)
(E) Treatment with TARE in patients intolerant of chemotherapy or patients considered not suitable for systemic therapy	2 (1.8)	1 (3.0)	–
(F) Other	6 (5.3)	2 (6.1)	14 (36.8)

## Methods

### Study Design/Setting

Patients for this 200-patient interim analysis were enrolled from 11 centres between August 2017 and October 2019, and follow-up data were collected until June 2020. All hospitals in France that had performed TARE with SIR-Spheres or were preparing to perform their first treatment were invited to participate. Hence, 31 sites were invited to participate, of which 4 declined participation, 4 were in the contracting process at the time of this analysis and 22 sites participated. Data were monitored by CIRSE remotely every 3 months to verify data quality and completion, and to identify any issues with patient inclusion and data collection. This study was performed in accordance with the Declaration of Helsinki and Good Clinical Practice Guidelines and was approved by the CPP SUD-EST II French Ethics Committee (N° ID-RCB: 2017-A01003-50). Since this study is an offspring of the European observational study CIRT [9] and closely corresponds to its methodology, please refer to the published methodology of CIRT for a more detailed description of methods and outcome measures [8].

### Patients

Patients were required to be at least 18 years old and to have primary or metastatic liver cancer intended to be treated with TARE (using SIR-Spheres) and to have signed an informed consent form. There were no further specified inclusion or exclusion criteria. Since the aim of the study was to capture all patients treated with SIR-Spheres in France, patients included in other trials were permitted to be enrolled. As a result, some intrahepatic cholangiocarcinoma patients of the present study were treated within the SIRCCA trial (NCT 02807181), but there were no patients in common with the European CIRT registry [9]. All aspects related to treatment and follow-up were performed at the discretion of the treating physicians. It was recommended to perform follow-ups every 3 months, for a period of at least 24 months.

### Outcomes and Data Sources

The primary outcome was defined as the real-life clinical application of TARE in order to assess at which stage of the cancer continuum of care the therapy was utilised. Secondary endpoints covered in this interim analysis were safety data and health-related quality of life (HRQOL) [12, 13]. For safety, adverse events (AE) and abnormal laboratory findings were graded according to the Cancer Institute’s Common Terminology Criteria for Adverse Events (CTCAE) v4.03. Adverse events were classified into three main groups; peri-procedural AE (on the same day of the treatment), AE within 30 days after treatment and AE after more than 30 days after treatment.

HRQOL was assessed using the EORTC QLQ-C30 questionnaire, as well as the QLQ-HCC18 Module for patients with hepatocellular carcinoma (HCC) [14]. Questionnaires were to be filled before, within one week after treatment and the first follow-up. Median first follow-up was at 13 weeks (IQR = 3).

For the HRQOL analysis (according to version 3 of the EORTC QLQ-C30 Scoring Manual (2001) and version 2 of the EORTC QLQ-HCC18 Scoring Manual), only patients with data available for all three timepoints (before treatment, within one week after treatment, and the first follow-up) were included, as a “per protocol” analysis. Scores for global health, functionality and symptoms were calculated by linearly transforming the mean of the respective raw scores on a scale of 0 to 100. For the global health and the functional score, a high score indicated high health and for the symptoms and the HCC18 Module a low score indicates few symptoms and better quality of life.

### Bias

In order to reduce patient selection bias, participation in the observational study was contractually required to be offered to all patients with primary or secondary liver tumours treated with TARE using SIR-Spheres. Furthermore, in order to assess patient coverage, case logs listing the number of patients treated versus number of patients actually enrolled in the study at each centre were collected quarterly from each of the 22 participating centres (please refer to supplementary Table 1 for more information).

### Statistical Methods

Baseline, treatment data and the secondary endpoint of safety were presented using summaries and descriptive statistics. For continuous data median, minimum and maximum were shown. To analyse categorical data, counts and percentages were used.

HRQOL data were plotted using RStudio under R 3.6.1. For both questionnaires, changes of more than 10 points were considered clinically relevant [15].

## Results

### Data Collection and Distribution of Patients

This interim report includes 200 patients, enrolled between August 2017 and October 2019. Treatment data were available for 98.5% and follow-up data for 93% of the patients, respectively. 11 centres actively contributed patient data, whereas another 11 centres performed no or very few cases and did not provide patient data. Patient numbers differed significantly between centres. In this interim analysis, a vast majority of 81.5% (*n* = 163) were treated at three sites; moreover, 54.5% (*n* = 109) of the overall patient population was contributed by one site. Median number of patients enrolled per site was 8.

With case logs collected quarterly from all 22 sites involved in the study, it was determined that the achieved coverage of all patients treated at participating sites was 84%. When disregarding one centre, the patient coverage achieved was 91% (please refer to supplementary Table 1 for more information).

### Patient Characteristics

Median age was 66 years (range 19–92), 140 (70%) patients were male. Of all 200 patients, HCC represented the majority (114, 57%) of cases, followed by mCRC (38, 19%) and intrahepatic cholangiocarcinoma (ICC; 33, 16.5%). Smaller cohorts were neuroendocrine tumour (NET; 5, 2.5%), and other malignancies (10, 5%, see Table 2).

**Table 2:** Patient demographics and treatments before and after TARE

Patient demographics *n* (%)	
Male	140 (70)
Age (median) (19, 92)	66
*Disease characteristics*	
Primary liver cancer	151 (75.5)
Secondary/metastatic liver cancer	49 (24.5)
*Primary tumours*	
HCC	114 (75.5)
ICC	33 (21.8)
Other	4 (2.7)
*Metastatic liver cancer*	
CRC	38 (77.5)
NET	5 (10.2)
Gastric cancer	1 (2)
Lung cancer	1 (2)
Other (Prostate cancer, missing)	4 (8.2)

Prior hepatic procedures	HCC *n* = 114 (%)	ICC *n* = 33 (%)	mCRC *n* = 38 (%)
Yes (subjects with at least one prior hepatic procedure)	61 (53.5)	2 (6.1)	18 (47.4)
No	53 (46.5)	31 (93.9)	20 (52.6)

Surgery	HCC *n* = 16 (14.0)	ICC *n* = 1 (3.0)	mCRC *n* = 9 (23.7)
Liver surgery	13 (11.4)	1 (3.0)	9 (23.7)
Open	9 (7.9)	1 (3.0)	4 (10.5)
Laparoscopy	4 (3.5)	–	2 (5.3)
Liver transplant	3 (2.6)	–	–

Ablation	HCC *n* = 17 (14.9)	ICC *n* = 0 (0)	mCRC *n* = 4 (10.5)
Radiofrequency Ablation	9 (7.9)	–	2 (5.3)
Microwave Ablation	10 (8.8)	–	1 (2.6)

Intra-arterial treatments	HCC *n* = 51 (44.7)	ICC *n* = 1 (3.0)	mCRC *n* = 4 (10.5)
Chemoembolization (TACE)	47 (41.2)	–	2 (5.3)
*Conventional TACE*	45 (39.5)	–	1 (2.6)
*Drug-eluting TACE*	2 (1.8)	–	1 (2.6)
Hepatic-arterialchemotherapy	–	–	2 (5.3)
Bland embolization	1 (0.9)	–	–
Vascular other	2 (1.8)	1 (3.0)	–

Radiation therapy (EBRT, Other)	HCC *n* = 1 (0.9)	ICC *n* = 0 (0)	mCRC *n* = 0 (0)
Other (cyberknife)	1 (0.9)	–	–

Prior systemic chemotherapy	HCC *n* = 16 (14)	ICC *n* = 13 (40)	mCRC *n* = 34 (90)
Hepatic Procedures after TARE	HCC *n* = 114 (%)	ICC *n* = 33 (%)	mCRC *n* = 38 (%)
Yes (Subjects with at least one hepatic procedure after TARE)	24 (21.0)	3 (9.1)	2 (5.3)
No	82 (71.9)	28 (84.8)	31 (81.6)
Unknown/data not available	8 (7.1)	2 (6.1)	5 (13.1)

Surgery	HCC *n* = 4 (3.5)	ICC *n* = 3 (9.1)	mCRC *n* = 1 (2.7)
Liver surgery	1 (0.9)	3 (9.1)	1 (2.7)
Open	1 (0.9)	1 (3.0)	1 (2.7)
Laparoscopy	–	2 (6.1)	–
Liver transplant	3 (2.7)	–	–

Ablation	HCC *n* = 4 (3.5)	ICC *n* = 0 (0)	mCRC *n* = 0 (0)
Radiofrequency ablation	1 (0.9)	–	–
Microwave ablation	2 (1.8)	–	–
Irreversible electroporation	1 (0.9)	–	–

Intra-arterial treatments	HCC *n* = 22 (%)	CC *n* = 0 (0)	mCRC *n* = 1 (2.7)
Chemoembolization (TACE)	20 (17.5)	–	1 (2.7)
Conventional TACE	16 (14.0)	–	–
Drug-eluting TACE	4 (3.5)	–	–
Hepatic-arterial chemotherapy	1 (0.9)	–	–
Bland embolization	1 (0.9)	–	–
Radiation therapy (TARE and EBRT)	HCC *n* = 2 (1.8) I	CC *n* = 0 (0)	mCRC *n* = 0 (0)
Systemic chemotherapy after TARE	HCC*n* = 28 (24.5)	ICC *n* = 22 (66.7)	mCRC *n* = 25 (65.7)

*EBRT* External beam radiation therapy

More than half of the HCC patients (53.5%) underwent liver-directed therapies before entering the study, mainly conventional transarterial chemoembolization (TACE; 39.5%) and 14% prior systemic chemotherapy.

93.9% of ICC patients received no surgery or ablation prior to TARE, but 40% received prior systemic chemotherapy. While the majority (84.8%) of these patients received no additional liver-directed treatment after TARE, 66.7% received systemic chemotherapy (Table 2).

In the mCRC cohort, 23.7% of patients received liver surgery before TARE and 10.5% received tumour ablation. Only 10% chemotherapy-naïve patients were encountered in the mCRC cohort, and 65.7% of patients received systemic chemotherapy treatment after TARE.

### Treatment Intent

Regarding the primary outcome of CIRT-FR, 37.7% of HCC (*n* = 43/114) and 54.5% of ICC (*n* = 18/33) patients underwent TARE with SIR-Spheres as a first-line treatment (Table 1). 43.8% (*n* = 50/114) HCC, 3% (*n* = 1/33) ICC and 5.2% (*n* = 2/38) mCRC patients received the treatment after previous liver-directed interventional radiological procedures or liver surgery in the absence of prior chemotherapy and post-hepatic procedures. A total of 52.6% (*n* = 20/38) of mCRC patients, 24.2% (*n* = 8/33) of ICC and 6.1% (*n* = 7/114) of HCC patients received TARE treatment after previous first-line systemic therapy.

### Tumour and Treatment-Related Characteristics

Please refer to Tables 3 and 4 for detailed information.

**Table 3:** Tumour location and Tumour and Liver volumes

Liver tumour location	HCC *n* = 114 (%)	ICC *n* = 33 (%)	mCRC *n* = 38 (%)
Left	22 (19.3)	6 (18.2)	3 (7.8)
Right	58 (50.9)	14 (42.4)	7 (18.4)
Bilobar	33 (28.9)	12 (36.4)	26 (68.4)
Unknown/data not available	1 (0.9)	1 (3.0)	2 (5.4)
**Number of liver tumours**			
1	46 (40.3)	20 (60.6)	3 (7.9)
2	16 (14.0)	2 (6.1)	3 (7.9)
3	16 (14.0)	2 (6.1)	4 (10.5)
4	8 (7.0)	–	2 (5.3)
5	4 (3.5)	–	3 (7.9)
> 5	7 (6.1)	3 (9.1)	8 (21.0)
Uncountable	15 (13.1)	5 (15.2)	11 (28.9)
Unknown/data not available	2 (1.8)	1 (2.9)	2 (5.3)
**Liver and tumour volumes (mL)**			*n* = 195 Median (IQR)
Left lobe volume (mL) *n* = 55			638 (460.5)
Left tumour volume (mL) *n* = 35			60 (122.3)
Right lobe volume (mL) *n* = 54			1104.5 (623.4)
Right tumour volume (mL) *n* = 42			136 (341.5)
Total liver volume (mL) *n* = 129			1765 (910)
Total tumour volume (mL) *n* = 128			129.5 (363)
**Tumour volume categories (mL)**			*n* = 197 (%)
1–50			41 (20.8)
51–100			37 (18.8)
101–200			30 (15.2)
201–500			43 (21.8)
501–1000			20 (10.2)
1001–2500			13 (6.6)
Unknown/data not available			13 (6.6)
**Liver cirrhosis**			
Yes	81 (71.0)	4 (12.1)	1 (2.7)
Alcohol	20 (24.7)	3 (75)	1 (100)
Hepatitis B	2 (2.5)	–	–
Hepatitis C	19 (23.5)	1 (25)	–
NAFLD	18 (22.2)	–	–
Alcohol + Hepatitis C	6 (7.4)	–	–
Alcohol + NAFLD	9 (11.1)	–	–
Other and unknown	7 (8.6)	–	–
No	33 (28.9)	28 (84.8)	35 (92.1)

Portal vein thrombosis (PVT)	HCC *n* = 114 (%)	ICC *n* = 33 (%)	mCRC *n* = 38 (%)
Patent	79 (69.3)	28 (84.8)	33 (86.8)
Segmental	26 (22.8)	2 (6.1)	2 (5.3)
Lobar	5 (4.4)	–	–
Main	2 (1.8)	2 (6.1)	–
Unknown/data not available	2 (1.8)	1 (3.0)	3 (7.9)

*NAFLD *non-alcoholic fatty liver disease

**Table 4:** Treatment procedure and administration

TARE treatment target	Centre	HCC *n* = 114 (%)	ICC *n* = 33 (%)	mCRC *n* = 38 (%)
Whole liver (single catheter)	1	1 (0.9)	–	–
	2	–	–	4 (10.5)
	3	–	–	1 (2.6)
	6	–	–	2 (5.3)
Whole liver (split administration/single session)	2	1 (0.9)	3 (9.1)	5 (13.2)
	3	–	1 (3.0)	–
	6	–	–	4 (10.5)
	8	–	–	1 (2.6)
Whole liver (sequential lobar/two sessions)	3	4 (3.5)	–	–
	5	2 (1.8)	–	1 (2.6)
Right lobe	1	45 (39.5)	15 (45.5)	5 (13.2)
	2	4 (3.5)	–	3 (7.9)
	3	6 (5.3)	–	–
	4	3 (2.6)	2 (6.1)	–
	5	2 (1.8)	1 (3.0)	2 (5.3)
	6			2 (5.3)
	7	2 (1.8)	–	1 (2.6)
	8	1 (0.9)	–	–
	10	1 (0.9)	–	–
Left lobe	1	18 (15.8)	5 (15.2)	1 (2.6)
	2	4 (3.5)	–	1 (2.6)
	3	5 (4.4)	–	1 (2.6)
	4	2 (1.8)	1 (3.0)	–
	5	–	–	1 (2.6)
	9	1 (0.9)	–	–
Segmental	1	4 (3.5)	3 (9.1)	–
	2	4 (3.5)	1 (3.0)	1 (2.6)
	3	1 (0.9)	–	–
	7	1 (0.9)	–	–
Unknown/data not available	1	1 (0.9)	–	–
	7	–	1 (3.0)	2 (5.3)
	11	1 (0.9)	–	–
**Methodology for determining the dose**				
BSA		12 (10.5)	5 (15.2)	19 (50.0)
Modified BSA		19 (16.7)	1 (3.0)	5 (13.2)
Partition model		78 (68.4)	26 (78.8)	8 (21.0)
Other		2 (1.8)	–	–
Unknown/ data not available		3 (2.6)	1 (3.0)	6 (15.8)

Prescribed/delivered activity				*n* = 231 (%)
Median prescribed activity whole (*n* = 126)				1 GBq
Unknown/data not available				3
Median prescribed activity right, GBq (*n* = 56)				1.2 GBq
Unknown/data not available				18
Median prescribed activity left, GBq (*n* = 40)				0.7 GBq
Unknown/data not available				34
**Delivered activity within 90% of the prescribed activity** ***n*** **= 197 (%)**				
Yes				188 (95.4)
No				3 (1.5)
Unknown/data not available				6 (3.0)
**Number of administrations delivered** *n* **= 197 (%)**				
1				59 (29.9)
2				24 (12.2)
3				3 (1.5)
Unknown/data not available				111 (56.3)
BSA, body surface area; GBq, gigabecquerel				

### Safety

Peri-procedural adverse events were reported during treatment for 8 (4%) patients, and 189 (94.5%) patients did not present with peri-procedural adverse events (see Table 5).

**Table 5:** Peri-interventional adverse events (AE) and AEs summary

	Peri-interventional AEs		< 30 days		> 30 days	
	Overall AE *n* = 9 (%)	AEs Grade 3 or 4 *n* = 6 (%)	Overall AE *n* = 42 (%)	AEs Grade 3 *n* = 6 (%)	Overall AE *n* = 382 (%)	AEs Grade 3 or 4 *n* = 36 (%)
Abdominal pain	4 (44.4)	4 (66.7)	2 (4.8)	–	50 (13.1)	4 (11.1)
Fatigue	–	–	11 (26.2)	–	91 (23.8)	8 (22.2)
Fever	–	–	–	–	9 (2.4)	–
Nausea	–	–	4 (9.5)	–	16 (4.2)	–
Vomiting	1 (11.1)	1 (16.7)	4 (9.5)	–	6 (1.6)	1 (2.8)
RE Induced Gastritis	–	–	1 (2.4)	–	–	–
Gastritis	–	–	–	–	2 (0.5)	–
RE Induced GI Ulceration	–	–	1 (2.4)	–	–	–
GI Ulceration	–	–	1 (2.4)	1(16.7)	3 (0.8)	2 (5.6)
REILD	–	–	1 (2.4)	1 (16.7)	–	–
Radiation Pneumonitis	–	–	–	–	–	–
Radiation Cholecystitis	–	–	–	–	–	–
Radiation Pancreatitis	–	–	–	–	–	–
Ascites	–	–	–	–	16 (4.2)	–
Dyspnoea	–	–	3 (7.1)	2 (33.3)	7 (1.8)	1 (2.8)
Hepatic enteropathy	–	–	–	–	8 (2.1)	4 (11.1)
Neuropathy	–	–	–	–	9 (2.4)	1 (2.8)
Hand–foot syndrome	–	–	–	–	13 (3.4)	1 (2.8)
Anorexia	–	–	1 (2.1)	–	15 (4.0)	4 (11.1)
Constipation	–	–	–	–	9 (2.4)	–
Diarrhoea	–	–	–	–	34 (8.9)	1 (2.8)
Anaemia	–	–	–	–	15 (4.0)	1 (2.8)
Jaundice	–	–	2 (4.8)	1 (16.7)	4 (1.0)	–
Oedema	–	–	3 (7.2)	1 (16.7)	3 (0.8)	–
**Less common AEs**	4 (44.4)	1 (16.7)	8 (19.1)	–	72 (18.8)	8 (22.2)
Bleeding	4 (44.4)	1 (16.7)	2 (4.8)	–	5 (1.3)	–
Liver and portal system	–	–			12 (3.2)	2 (5.6)
Cardio-pulmonary	–	–	–	–	5 (1.3)	–
Neurological, pain, and other sensitive disorders	–	–	–	–	15 (3.9)	–
General	–	–	4 (9.5)	–	9 (2.4)	1 (2.8)
Digestive	–	–	1 (2.4)	–	9 (2.4)	–
Analytical	–	–	–	–	4 (1.0)	1 (2.8)
Cutaneous complications	–	–	1 (2.4)	–	7 (1.8)	1 (2.8)
Musculoskeletal disorders	–	–	–	–	3 (0.8)	–
Pancreatitis	–	–	–	–	2 (0.5)	2 (5.6)
Renal and fluid balance	–	–	–	–	1 (0.3)	1 (2.8)

	Peri–interventional		< 30 days		> 30 days	
	Overall	Grade 3 or 4	Overall	Grade 3	Overall	Grade 3 or 4
Subjects with at least one AE* n* = 200 (%)	8 (4)	4 (2)	24 (12)	3 (1.5)	122 (61)	28 (14)

*REILD,*radioembolization-induced liver disease;* GI* gastrointestinal

189 (94.5%) patients had no severe peri-interventional AEs. For 3 (1.5%) patients, no information was collected. Subjects with at least one peri-interventional AEs were 8 (4%), of which 4(2%) had grade 3. There were 4 (2%) other peri-interventional AEs reported in 4 patients (three vascular minor and one blood transfusion) no grading for these four AEs was provided. A total of 42 AEs were observed in 24 patients within 30 days following treatment of which 6 were grade 3 (GI Ulceration, REILD, jaundice, oedema and dyspnoea). No AE grade 4 or 5 were observed during the first 30 days after treatment

Within 30 days following treatment, 24 (12%) of patients experienced at least one AE and a total of 42 AE, with 82% of graded AEs reported to be mild to moderate (i.e. grade 1 or 2). Six grade 3 AEs in three patients (GI ulceration, radioembolization induced liver disease (REILD), jaundice, oedema and dyspnoea) were reported. No AE grade 4 or 5 was reported during the first 30 days.

Overall, 122 (61%) patients experienced at least one AE more than 30 days following treatment and a total of 382 AE, with 83% of graded AEs being reported as moderate. 36 (9.4%) AEs in 28 (14%) patients were grade 3 or 4, and no AEs grade 5 was reported. 168 (44%) AEs were not graded. AEs of special relevance or interest for TARE are listed in Table 5 together with commonly reported AEs. The remaining reported AEs are categorised in Table 5, section ‘’Less common AEs’’. No radiation induced pneumonitis, cholecystitis or pancreatitis were reported in the cohort.

### Quality of Life

**Fig. 1:**
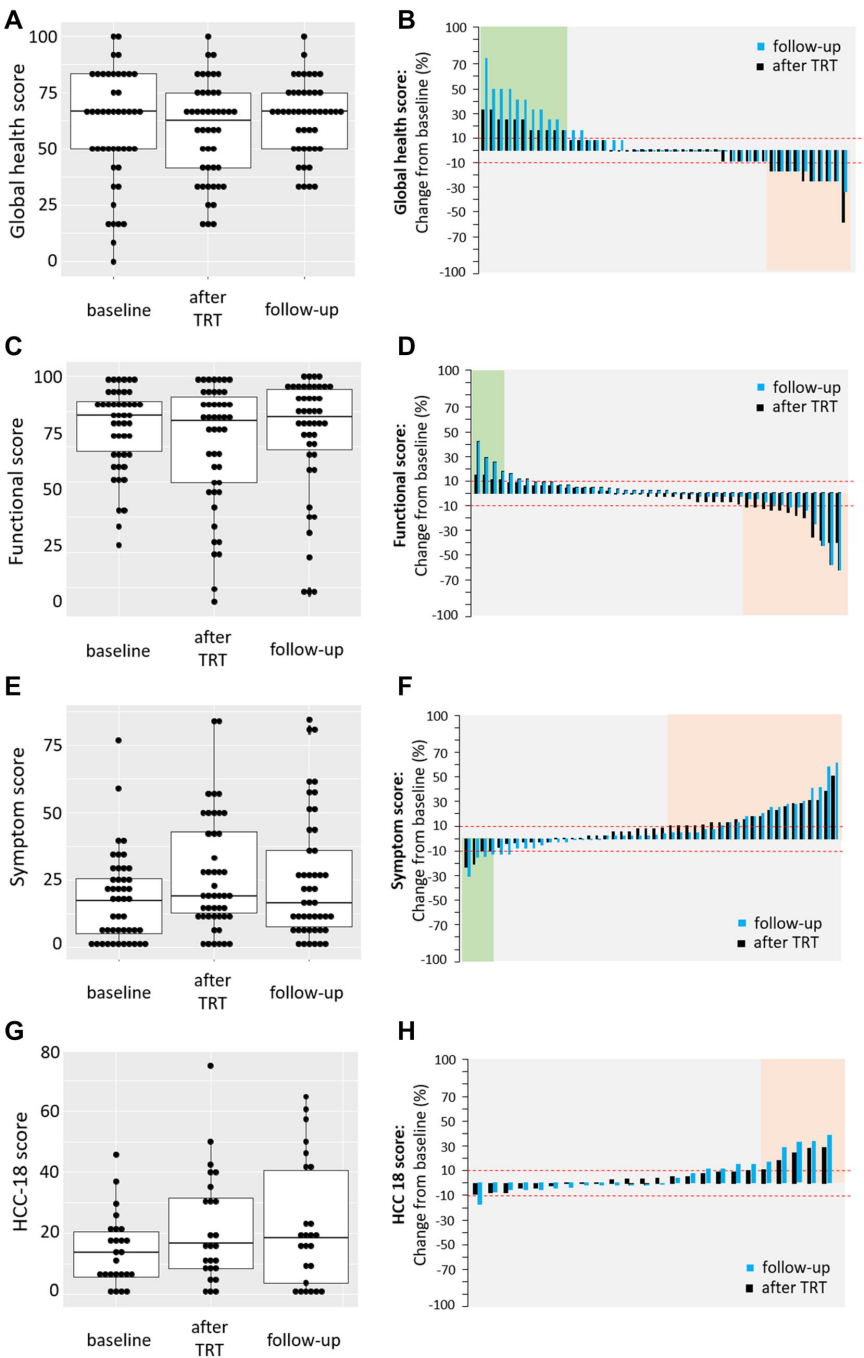
Health-related quality of life over time (EORTC-QLQ 30). Shows HRQOL score of 46 patients (**A**-**F**) and 25 patients (**G**,** H**) collected at baseline (before the first treatment), right after treatment (within 1 week later) and at the first follow-up (13 weeks after treatment on average) by analysing global health (**A**,** B**), functionality (**C**,** D**), symptoms (**E**,** F**) and HCC18 module (**G**,** H**) score according to EORTC-QLQ30 version 3.0 and the EORTC QLQ-HCC18 version 2.0. For the global health and the functional score, a high score indicates high health and for the symptoms and HCC18 scale a low score indicates few symptoms and better HRQOL, respectively. For general comparisons between baseline to right after treatment and baseline to the first follow-up, boxplots were used (**A**,** C**,** E**,** G**). The difference between baseline to right after treatment (black) and baseline to first follow-up (light colour) was plotted for individual patients using a waterfall diagram (**B**, **D**, **F**, **H**). Cut-offs (dashed line) for clinically significant improvement were set at + 10 for global health, functional score, − 10 for symptoms score, HCC18 module and at -10 for global health, functional score and + 10 for symptoms score, HCC18 module for deterioration

Health-related quality of life was assessed using the EORTC QLQ-C30 questionnaire at baseline (range 99 – 0 days before treatment), within one week after treatment and on the first follow-up at an average of 13 weeks (range 5–19) following treatment for the 23% of patients that had questionnaires available for all three timepoints. Figure 1A, C and E shows that the median HRQOL at baseline compared to after treatment and the first follow-up remains stable for the global health score (66.7% vs. 62.5% and 66.7%), for the functional score (86.2% vs. 84.4% and 83.3%) and for symptom score (17.9% vs. 20.5% and 15.4%).

At a patient level, when comparing baseline to after treatment, improvement indicated by an increase in more than 10 points was seen in 23.9% for global health and 8.6% for functional and a decline by more than 10 points in 21.7 and 26%, respectively (Fig. 1B, D, black colour). A decline in symptoms and thus improvement of HRQOL was seen in 8.6% vs 45.6% showing increased symptoms (Fig. 1F, black colour). The number of patients with increased or decreased HRQOL did not change by much when comparing the first follow-up to baseline (Fig. 1B, D, F light colour). Quality of life status was distributed evenly across indications (data not shown).

With regards to the HCC18 Module, information was provided for 25 (22%) HCC patients at baseline at an average of 13 days before treatment (range 0 to 43), immediately after treatment and on the first follow-up at an average of 13 weeks (range 5.4 to 18.4) following treatment. The HCC18 global health sub-score (Fig. 1G) worsened across baseline (14.4%), treatment (21.3%) and the first follow-up (22.5%). When comparing to baseline, at a patient level (Fig. 1H), the HCC 18 score improved by more than 10 points in 4% of patients at follow-up 1 (light colour) compared to baseline and declined by more than 10 points in 36% at follow-up 1 (light colour) and 20% after treatment (black) when compared to baseline.

## Discussion

In contrast to many other medical devices used for local ablative therapies, SIR-Spheres microspheres have an abundance of clinical data-available from randomised-controlled trials that speak to the general safety and effectiveness of the treatment [10, 11, 16–18]. While one of the randomised-controlled hallmark trials assessing this treatment modality was conducted in France [10], real-world data were still lacking.

Despite the observational, real-world setting of CIRT-FR, the observed HCC patient populations were similar to the inclusion criteria of the SARAH trial. For SARAH, patients were eligible if they had an Eastern Cooperative Oncology Group (ECOG) performance status of 0 or 1, Child–Pugh liver function class A or B and locally advanced HCC. Similar to this, 92% of the CIRT-FR HCC cohort had an ECOG performance status of 0 or 1 and 77% had Child–Pugh liver function class A or B (data not shown). However, the CIRT-FR HCC patient population presented with less locally advanced HCC when compared to the SARAH trial (63% portal vein thrombosis in SARAH vs 29% in CIRT-FR).

This interim analysis constitutes a representative study on the current real-life application of TARE using SIR-Spheres in France. Based on information collected from all 22 participating sites, 84% (91% disregarding one uncompliant site) of all patients treated were included in the study. Furthermore, information on institutions using the treatment was provided by Sirtex (Sirtex Medical Europe GmbH, Bonn, Germany) before the start of the study and updated regularly. 31 centres using SIR-Spheres therapy in France were invited to participate, of which 11 actively contributed patient data and the remainder provided case logs. There are two distinct considerations between the representativeness at the site-level (i.e. how well the overall data describes real-life clinical practice in each of the participating sites) and representativeness at the national level for France overall (i.e. how well the overall data describes real-life clinical practice with SIR-Spheres therapy in France at the moment). Even though one hospital is more highly represented in the data and overall results cannot be considered to represent real-life clinical practice at the other sites, the presented data are representative of real-life clinical practice in France. Representativeness of data in France is reflected by the high coverage of treated patients achieved in this study in relation to the low number of non-participating sites using SIR-Spheres therapy.

An essential aspect of evaluation of yttrium-90 microspheres usage in real-life clinical practice is to understand at which stage of the continuum of care this treatment is utilised. In HCC, TARE was mainly used following an interventional liver-directed procedure (48%) or liver surgery (14%). Additionally, a large proportion of patients with HCC were also treated with TARE in a first-line treatment setting (37.7%), of which 35% had portal vein thrombosis (28% segmental and 7% lobar). Furthermore, for the same cohort 65% of patients had 1 or 2 tumours, 21% of patients 3 to 9 tumours and 14% of patients had more than 10 or an uncountable number of tumours and median tumour volume for the whole group was 240 mL (IQR = 773) (data not shown). When comparing dosimetry values to the SARAH trial (median 1000 MBq), they were found to be similar to those reported in CIRT-FR (median 952.5 MBq) Considering that SIR-Spheres therapy was only reimbursed for the treatment of HCC for 7 of the 25 months of patient enrolment covered in this interim analysis, it stands to reason that sites anticipated the reimbursement recommendation and that reimbursement is not a deciding factor for treatment administration when a treatment is considered clinically effective. When no reimbursement was available for SIR-Spheres therapy reimbursement for HCC or other indications, treatment costs were covered by the hospitals.

Furthermore, 33 ICC patients were included in CIRT-FR in the absence of reimbursement for TARE in this entity. The majority of these patients might have been enrolled in the SIRCCA trial (NCT 02807181) evaluating TARE prior to chemotherapy treatment in chemotherapy naïve ICC patients. This also puts into perspective the unusually high number of chemotherapy-naïve patients (60%) encountered in this cohort, which is in contrast to published literature [19] and to what was found in CIRT [9].

Rates of ~ 20% grade ≥ 3 AEs have been reported in randomised-controlled trials on TARE [10, 11, 16–18, 20]. In the real-life setting, the European CIRT observational study [9] reports rates of 2.5% grade 3–4 AEs and the BSIR registry found 8% of all AEs to be above grade 3 in mCRC patients [21] as well as ICC patients [19]. The low rate of AEs < 30 days following treatment (12% of the patients) presented in this study in addition to the low frequency of patients experiencing at least one treatment-related Grade ≥ 3 SAEs in the overall patient cohort (16%), confirms the notion that TARE can be considered as a safe treatment alternative [22–26].

Studies have found a lower impact on HRQOL when using TARE with yttrium-90 microspheres compared to other loco-regional treatments, such as TACE [16] or systemic chemotherapy [10, 27]. Using the EORTC QLQ C-30 questionnaire, Vilgrain et al. found a persistent mean global health status sub-score of about 60% over a period of 12 months after TARE [10]. Using the Functional Assessment of Cancer Therapy Hepatobiliary (FACT-Hep) survey, Kolligs et al. [16] and Salem et al. [27] also observed relatively stable HRQOL scores over a 12- and 4-week period, respectively. While presented data are presently limited to the first follow-up, our mean global health sub-score of 60.2% indicates that HRQOL remains relatively constant when compared to baseline (57.6% and 63.9% at 13 weeks; baseline 59.1%). It is therefore reassuring to see that HQOL observed in controlled trials holds true to the real-life clinical practice.

### Limitations

Substantial difficulties in QOL questionnaire collection over time were notorious. Only 23% of patients provided this information for all three timepoints required. While similar return rates are to be expected for the overall patient cohort, the bigger sample size will bolster the credibility of presented results, especially when utilising a method for imputing missing items from multi-item scales to deal with missing HRQOL data and analysing in conjunction with data from the European CIRT observational study [9]. Furthermore, a limitation based on the structure of the electronic data capture system is the high number of ungraded AEs (44%; AE grade was a non-mandatory data field), data which we hope to obtain from sites until the final analysis.

Another observed limitation is that more than half of all patients were provided by a single site. This might have considerably skewed results in favour of clinical practice conducted at that respective site. The final results of CIRT-FR will include a statistical analysis assessing the effect of the disproportionate contribution from one centre regarding patient enrolment.

### Outlook

CIRT-FR patient inclusion terminated in August 2020, and follow-up data will be collected for 2 years, until August 2022. Data on the entire study cohort as well as data on effectiveness will be published once data collection is complete. Furthermore, in order to provide an overview of real-life clinical practice across Europe, the data collected in CIRT-FR is foreseen to be analysed in relation with data collected by CIRT [9], and the observational study that gathered data on TARE with SIR-Spheres in Europe.

## Conclusion

Regarding the real-life clinical use of TARE in France, treatment intent varied across observed indications and was largely used as a palliative or first line treatment for HCC. Similarly, TARE was indicated to be used as a first line treatment for the majority of ICC patients. For mCRC patients, TARE treatment intent was as a second line or salvage therapy treatment for the majority of patients.

Overall, it is reassuring to see that data regarding safety and quality of life generated by randomised-controlled trials is reflected when assessing the real-world application of TARE in this interim analysis.

## Electronic supplementary material

Below is the link to the electronic supplementary material.

Supplementary file1 (DOCX 14 kb)

## Footnotes

1 HAS (2015). Avis de la CNEDiMTS sur SIR-Spheres. Retrieved March 2, 2017, from https://www.has-sante.fr/portail/jcms/c_2023879/fr/sir-spheres

2 HAS (2018). Avis de la CNEDiMTS sur SIR-Spheres. Retrieved October 7, 2019, from https://www.has-sante.fr/jcms/c_2896412/fr/sir-spheres

## References

[1] Golfieri R (2014). SIR-Spheres yttrium-90 radioembolization for the treatment of unresectable liver cancers. Hepatic Oncol.

[2] Mosconi C, Cappelli A, Pettinato C, Golfieri R (2015). Radioembolization with Yttrium-90 microspheres in hepatocellular carcinoma: Role and perspectives. World J Hepatol.

[3] Vogel A, Cervantes A, Chau I, et al. Hepatocellular carcinoma: ESMO Clinical Practice Guidelines for diagnosis, treatment and follow-up. Ann Oncol 2018;29:iv238–iv255. 10.1093/annonc/mdy308 10.1093/annonc/mdy308 30285213

[4] Van Cutsem E, Cervantes A, Adam R (2016). ESMO consensus guidelines for the management of patients with metastatic colorectal cancer. Ann Oncol.

[5] Memon K, Lewandowski RJ, Kulik L (2011). Radioembolization for primary and metastatic liver cancer. Semin Radiat Oncol.

[6] Salem R, Lewandowski RJ, Gates VL (2011). Research reporting standards for radioembolization of hepatic malignancies. J Vasc Interv Radiol.

[7] Galle PR, Forner A, Llovet JM (2018). EASL clinical practice guidelines: management of hepatocellular carcinoma. J Hepatol.

[8] Helmberger T, Arnold D, Bilbao JI, et al. Clinical application of radioembolization in hepatic malignancies: Protocol for a prospective multicenter observational study. J Med Internet Res. 2020;22: 10.2196/16296. 10.2196/16296 PMC7203613 32319960

[9] Helmberger T, Golfieri R, Pech M, et al. Clinical Application of TARE in Hepatic Malignances in Eurpoe: first results from the prospective multicentre observational study CIRSE Registry for SIR-Spheres Therapy (CIRT). Cardiovasc Interv Radiol 2020 (in press) 10.1007/s00270-020-02642-y PMC7728645 32959085

[10] Vilgrain V, Pereira H, Assenat E (2017). Efficacy and safety of selective internal radiotherapy with yttrium-90 resin microspheres compared with sorafenib in locally advanced and inoperable hepatocellular carcinoma (SARAH): an open-label randomised controlled phase 3 trial. Lancet Oncol.

[11] Chow PKH, Gandhi M, Tan SB (2018). SIRveNIB: Selective internal radiation therapy versus sorafenib in Asia-Pacific patients with hepatocellular carcinoma. J Clin Oncol.

[12] Fayers P, Bottomley A (2002). Quality of life research within the EORTC—The EORTC QLQ-C30. Eur J Cancer.

[13] NCI, NIH D (2009) Common Terminology Criteria for Adverse Events v4.0. NIH Publ 2009:0–71

[14] Blazeby JM, Currie E, Zee BCY (2004). Development of a questionnaire module to supplement the EORTC QLQ-C30 to assess quality of life in patients with hepatocellular carcinoma, the EORTC QLQ-HCC18. Eur J Cancer.

[15] Cocks K, King MT, Velikova G (2011). Evidence-based guidelines for determination of sample size and interpretation of the European organisation for the research and treatment of cancer quality of life questionnaire core 30. J Clin Oncol.

[16] Kolligs FT, Bilbao JI, Jakobs T (2015). Pilot randomized trial of selective internal radiation therapy vs. chemoembolization in unresectable hepatocellular carcinoma. Liver Int.

[17] Ricke J, Bulla K, Kolligs F (2015). Safety and toxicity of radioembolization plus Sorafenib in advanced hepatocellular carcinoma: analysis of the European multicentre trial SORAMIC. Liver Int.

[18] Salem R, Gordon AC, Mouli S, Hickey R, Kallini J, Gabr A, Mulcahy MF, Baker T, Abecassis M, Miller F, Yaghmai V, Sato K, Desai K, Thornburg B, Benson AB (2017). RE: Y90 radioembolization significantly prolongs time to progression compared with chemoembolization in patients with hepatocellular carcinoma. Gastroenterology.

[19] White J, Carolan-Rees G, Dale M (2019). Yttrium-90 transarterial radioembolization for chemotherapy-refractory intrahepatic cholangiocarcinoma: a prospective, observational study. J Vasc Interv Radiol.

[20] Salem R, Lewandowski RJ, Kulik L (2011). Radioembolization results in longer time-to-progression and reduced toxicity compared with chemoembolization in patients with hepatocellular carcinoma. Gastroenterology.

[21] White J, Carolan-Rees G, Dale M (2019). Analysis of a national programme for selective internal radiation therapy for colorectal cancer liver metastases. Clin Oncol.

[22] Gangi A, Shah J, Hatfield N (2018). Intrahepatic cholangiocarcinoma treated with transarterial Yttrium-90 glass microsphere radioembolization: results of a single institution retrospective study. J Vasc Interv Radiol.

[23] Bourien H, Palard X, Rolland Y (2019). Yttrium-90 glass microspheres radioembolization (RE) for biliary tract cancer: a large single-center experience. Eur J Nucl Med Mol Imaging.

[24] Khor AYK, Toh Y, Allen JC (2014). Survival and pattern of tumor progression with yttrium-90 microsphere radioembolization in predominantly hepatitis B Asian patients with hepatocellular carcinoma. Hepatol Int.

[25] Kim DY, Park BJ, Kim YH (2015). Radioembolization with Yttrium-90 resin microspheres in hepatocellular carcinoma: a multicenter prospective study. Am J Clin Oncol Cancer Clin Trials.

[26] Sangro B, Carpanese L, Cianni R (2011). Survival after Yttrium-90 resin microsphere radioembolization of hepatocellular carcinoma across Barcelona clinic liver cancer stages: a European evaluation. Hepatology.

[27] Salem R, Gilbertsen M, Butt Z (2013). Increased quality of life among hepatocellular carcinoma patients treated with radioembolization, compared with chemoembolization. Clin Gastroenterol Hepatol.

