# Supplementary material for: Short-term Safety and Quality of Life Outcomes Following Radioembolization in Primary and Secondary Liver Tumours: a Multi-centre Analysis of 200 Patients in France
Source: Cardiovasc Intervent Radiol. 2020 Sep 25;44(1):36–49. doi: 10.1007/s00270-020-02643-x (PMC7728633; doi:10.1007/s00270-020-02643-x)
Supplement: Supplementary file 1 — Supplementary file1 (DOCX 14 kb) [file 270_2020_2643_MOESM1_ESM.docx]

**Supplementary table 1.** Patient coverage and case log information

|  | **Total patients treated from contract signage** | **Enrolled in CIRT-FR** | **Not enrolled in CIRT-FR** |
| --- | --- | --- | --- |
| **Enrolling centres** | | | |
| 1 | 109 | 109 | 0 |
| 2 | 32 | 32 | 0 |
| 3 | 23 | 22 | 1 |
| 4 | 8 | 8 | 0 |
| 5 | 12 | 8 | 4 |
| 6 | 8 | 8 | 0 |
| 7 | 9 | 8 | 1 |
| 8 | 6 | 2 | 4 |
| 9 | 3 | 1 | 2 |
| 10 | 1 | 1 | 0 |
| 11 | 19 | 1 | 18 |
| **Non-enrolling centres** | | | |
| 1 | 2 | - | 2 |
| 2 | 0 | - | 0 |
| 3 | 3 | - | 3 |
| 4 | 0 | - | 0 |
| 5 | 1 | - | 1 |
| 6 | 0 | - | 0 |
| 7 | 1 | - | 1 |
| 8 | 0 | - | 0 |
| 9 | 0 | - | 0 |
| 10 | 0 | - | 0 |
| 11 | 0 | - | 0 |
